# Case report: Percoronary device closure of tortuous coronary artery fistula into left atrium

**DOI:** 10.3389/fcvm.2023.1106420

**Published:** 2023-02-14

**Authors:** Shi-Bin Sun, Zhongzheng Kong, Zeeshan Farhaj, Li Hongxin

**Affiliations:** ^1^Department of Cardiovascular Surgery, The First Affiliated Hospital of Shandong First Medical University and Shandong Provincial Qianfoshan Hospital, Shandong Engineering Research Center for Heart Transplant and Material, Jinan, China; ^2^Department of Cardiovascular Surgery, Shandong Provincial Qianfoshan Hospital, Weifang Medical University, Weifang, China

**Keywords:** coronary artery fistula, percoronary, device closure, minithoracotomy, occlusion

## Abstract

Surgical ligation and transcatheter occlusion are the mainstream for the treatment of coronary artery fistulas (CAFs). However, these techniques applied to tortuous and aneurysmal CAF, especially those draining into left-heart, have their known drawbacks. We report, a successful percoronary device closure of such CAF, originating from left main coronary artery and draining into left atrium, through a left subaxillary minithoracotomy. Through a puncture on the distal straight course, we occluded CAF exclusively under transesophageal echocardiography guidance. Complete occlusion was achieved. It’s a simple, safe, and effective alternative for tortuous, large, and aneurysmal CAFs draining into the left heart.

## Introduction

Coronary artery fistula (CAF) is a rare congenital heart disease. Surgical ligation and transcatheter device closure (TCC) are the mainstream for treatment of CAF (1–3). However, surgical ligation is associated with significant trauma, morbidity, discomfort, an unsightly scar and the use of cardiopulmonary bypass. The TCC is a preferable way of treatment, but is troublesome in highly tortuous, large, and aneurysmal CAF, especially those draining into the left heart chamber. We present an alternative and achievable technique of percoronary device closure (PDC) of CAF, which was tortuous, large, aneurysmal and draining into the left atrium (LA), under exclusive transesophageal echocardiography (TEE) guidance.

## Case presentation

**FIGURE 1:**
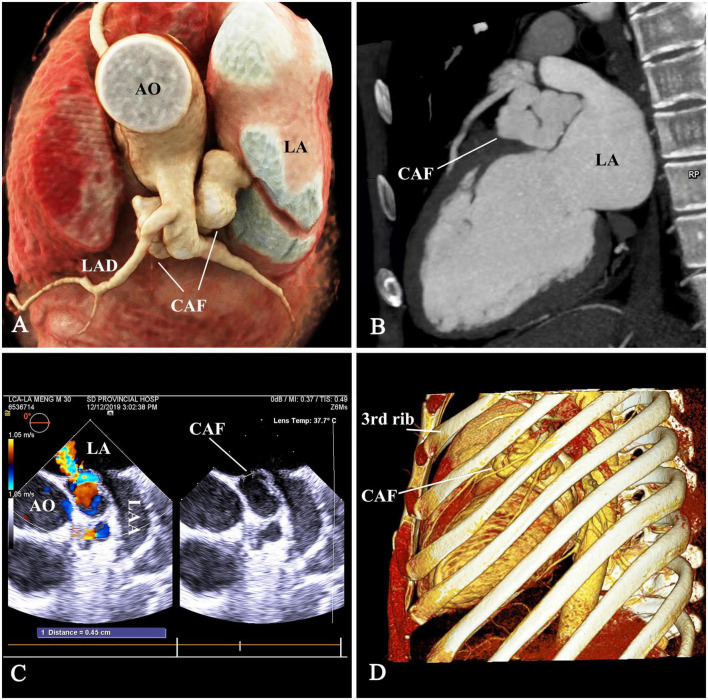
The anatomy of CAF. **(A,B)** The tortuous aneurysmal CAF originated from main coronary artery (MCA), made a 180° turn underneath the LAD and MCA, and coursed backward to drain into the LA. **(C)** The drainage opening measuring 4.5 mm, was located at the LA roof between the aortic root and LAA. **(D)** The thoracic computed-tomographic-angiography to determine the access. CAF, coronary artery fistula; AO, ascending aorta; LAD, left anterior descending branch; LA, left atrium; LAA, left atrial appendage.

A 32-year-old man was admitted with a left scapular pain and diagnosed with a CAF by TEE and computed tomographic angiography (CTA). The TEE showed the CAF originated from main coronary artery (MCA) and drained into the LA with a continuous left-to-left shunt. The maximum inner diameter of the CAF was 11 mm and the left ventricular end diastolic diameter was 59 mm. The CTA was performed to delineate the anatomy of the CAF (Figures 1A–C). A continuous murmur was detected in the parasternal left 2nd and 3rd intercostal space. Non-specific electrocardiogram abnormalities were found. A bone-reserved thoracic CTA was also performed before the procedure to determine the exact location of the incision and procedure planning (Figure 1D). The informed consent was obtained.

**FIGURE 2:**
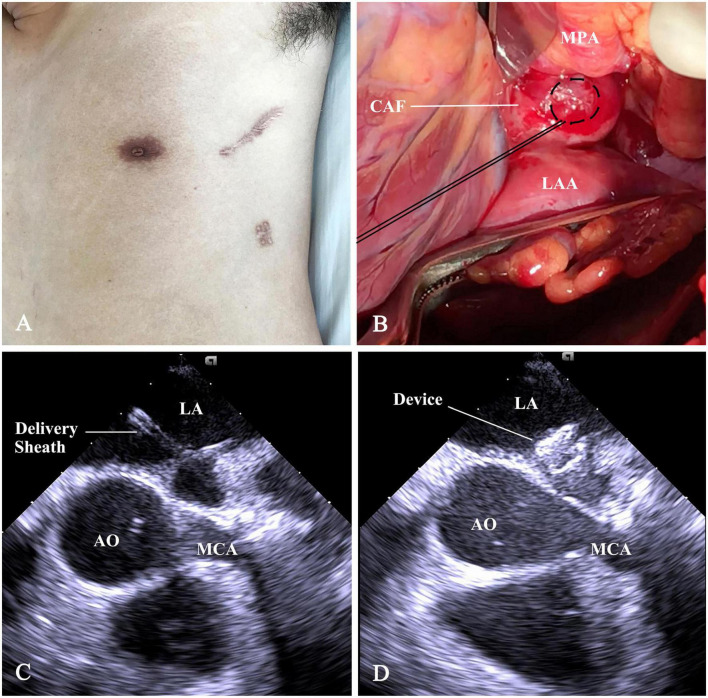
Schematic presentation of percoronary device closure of CAF. **(A)** The subaxillary incision. **(B)** The distal straight section of CAF was exposed between the main pulmonary artery (MPA), root of AO, and the LAA. A purse-string suture was placed on it. **(C)** Following a puncture in purse-string, a flexible guidewire was advanced into LA. Then, a 6F short delivery sheath was fed over the wire into the LA. **(D)** The device was deployed and positioned at the drainage orifice of the CAF. CAF, coronary artery fistula; AO, ascending aorta; LAA, left atrial appendage; LA, left atrium; MPA, main pulmonary artery; MCA, main coronary artery.

Under general anesthesia, the patient was placed in a right lateral position. A 7-cm subaxillary incision was made in the left 4th intercostal space. The pericardium was incised and cradled. An ordinary (size 8 mm) ventricular septal occluder (Starway Medical Technology, Inc, Beijing, China), connected with a stay-in suture (5-0 Polydioxanone, Ethicon, Somerville), was selected and retracted into the loading sheath. Figure 2 showed the steps of percoronary device closure of CAF. A 15-min occlusion test of CAF was well-tolerated; there was no evidence of myocardial ischemia. The occluder was scrutinized by using a push-pull maneuver repeatedly and released after satisfactory assessment. The device stay-in suture was removed before the purse-string suture snugly tied. The pericardium and incision were closed in layers with a drainage tube.

**FIGURE 3:**
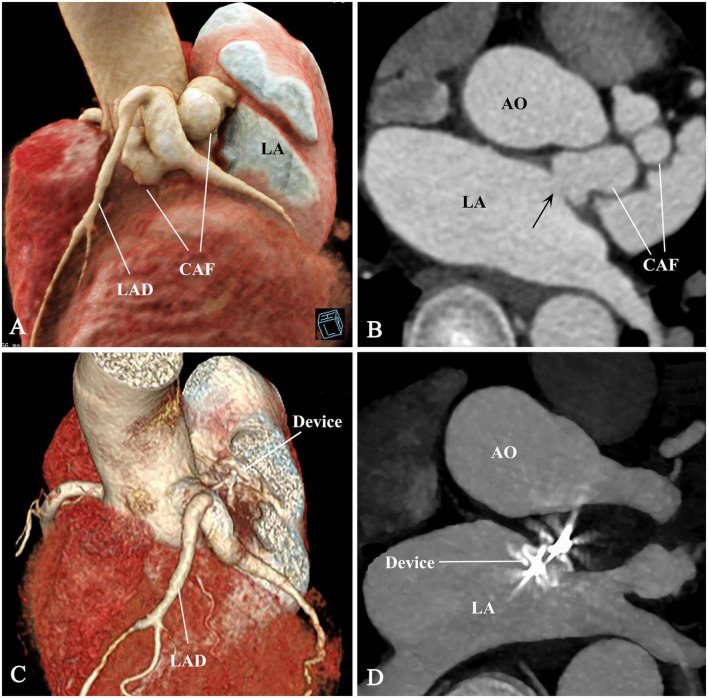
Pre- and post-procedural comparison of CAF. **(A–D)** The CAF disappeared and thrombogenesis occurred from distal to proximal segment of the CAF. CAF, coronary artery fistula; AO, ascending aorta; LAD, left anterior descending branch; LA, left atrium; arrow, fistulous drainage orifice.

Complete occlusion was achieved immediately after device release. Serial CKMB and troponin levels in the first 48 h were within normal limits. The patient recovered uneventfully and discharged 7 days postoperative. The murmur disappeared post-occlusion. Antiplatelet therapy was maintained on aspirin (100 mg/day) and clopidogrel (75 mg/day) for a year. Medical evaluations including electrocardiograms, TTE, CTA, and stress tests were reviewed during the follow-up period of 18 months. These records demonstrated an appropriate device position and complete occlusion of the CAF and no complications (Figures 3A–D). The Supplementary Figure 1 shows the device and stay-in-suture use.

## Comment

Coronary artery fistulas originating from the right coronary artery account for 50–60% of cases and often drain into the right heart (80%). Only 0.7% of cases originate from MCA and only 5–6% drain into the LA (1–3).

The common treatment for CAF is surgical and TCC. The choice of the technique depends on its morphology, course, tortuosity, and the presence of aneurysmal CAF.

The major advantages of TCC over surgery include the avoidance of cardiopulmonary bypass, median sternotomy and the related complications, short recovery time, lower morbidity, and improved cosmetic results. The TCC approaches are antegrade venous, retrograde arterial, or arteriovenous loop. If the drainage orifice locates in the right heart chamber and is easy to access, the antegrade venous approach is the preferred route, whereas if access is difficult, the arteriovenous loop method is a better choice (2, 3). Another access route for highly tortuous CAF, draining into the right heart, is a perventricular approach (4). However, TCC of fistulas, draining into the left heart chambers, is challenging and can only be achieved by retrograde arterial, or “arterioarterial loop” approach.

The proximal CAF arising from MCA is highly recommended to close, as this type of fistula has the risk of increased aneurismal dilatation and rupture. When the drainage site is located in the left-heart, the CAF is particularly tortuous (3, 5). It results in not only the well-known “coronary steal phenomenon,” but also a left-to-left shunt that increases a volume overload to the left heart.

Although coils can be delivered with a retrograde arterial approach through very small catheters and sheaths, the tortuous aneurismal CAF prevent the guidewire and sheath’s advancement and increase the risk of rupture. Additionally, the coil occlusion in large CAF might be unstable or incomplete. Mispositioning or proximal extension of the coils may result in obstruction of coronary branches and myocardial ischemia. Incomplete occlusion might raise concerns for bacterial endocarditis.

In this case, the CAF was large, aneurysmal, and tortuous. The distal segment, which was adjacent to ascending aorta, LA, MCA (Figures 1A, 2B) and in deep anatomic position, was exposed through minithoracotomy. Surgical ligation is almost unthinkable because of limited anatomic space and the risk of catastrophic bleeding. For TCC, there was not a feasible segment for positioning a double-disk device except the segment below the MCA and the drainage opening at the LA (Figures 1A, 3A). Device positioning below the MCA has the risk of impingement on the MCA. Device occlusion at the fistula drainage opening should be the best choice, although the drainage site is almost inaccessible for the delivery sheath due to the acute turn and tortuous course of the CAF.

The PDC provides a straighter catheter course and a good puncture angle, avoids potential damage to the adjacent structures and femoral artery. The puncture site is chosen at the distal straight course of CAF near the drainage opening, allowing larger catheters to be used. This technique is a simple, safe, and effective alternative therapy for tortuous, large, and aneurysmal CAFs draining into the left heart chamber.

## Data availability statement

The raw data supporting the conclusions of this article will be made available by the authors, without undue reservation.

## Ethics statement

The studies involving human participants were reviewed and approved by the Ethics Committee of the First Affiliated Hospital of Shandong First Medical University. The patients/participants provided their written informed consent to participate in this study.

## Author contributions

S-BS and ZK: conception, data collection, data analysis, interpretation, drafting the manuscript, and revision. ZF and LH: conception, drafting the manuscript, and critical revision. All authors contributed to the article and approved the submitted version.

## Supplementary material

Supplementary Figure 1 The device can be seen attached to the delivery cable and ready to be loaded in the delivery sheath. The polydioxanone stay-in-suture can be seen passed through the center of the device under the screw, then passed back through the sheath. The inset shows the closeup of the device with stay-in-suture.
