# Supplementary figures and images for: Case report: Percoronary device closure of tortuous coronary artery fistula into left atrium
Source: Front Cardiovasc Med. 2023 Feb 14;10:1106420. doi: 10.3389/fcvm.2023.1106420 (PMC9971947; doi:10.3389/fcvm.2023.1106420)

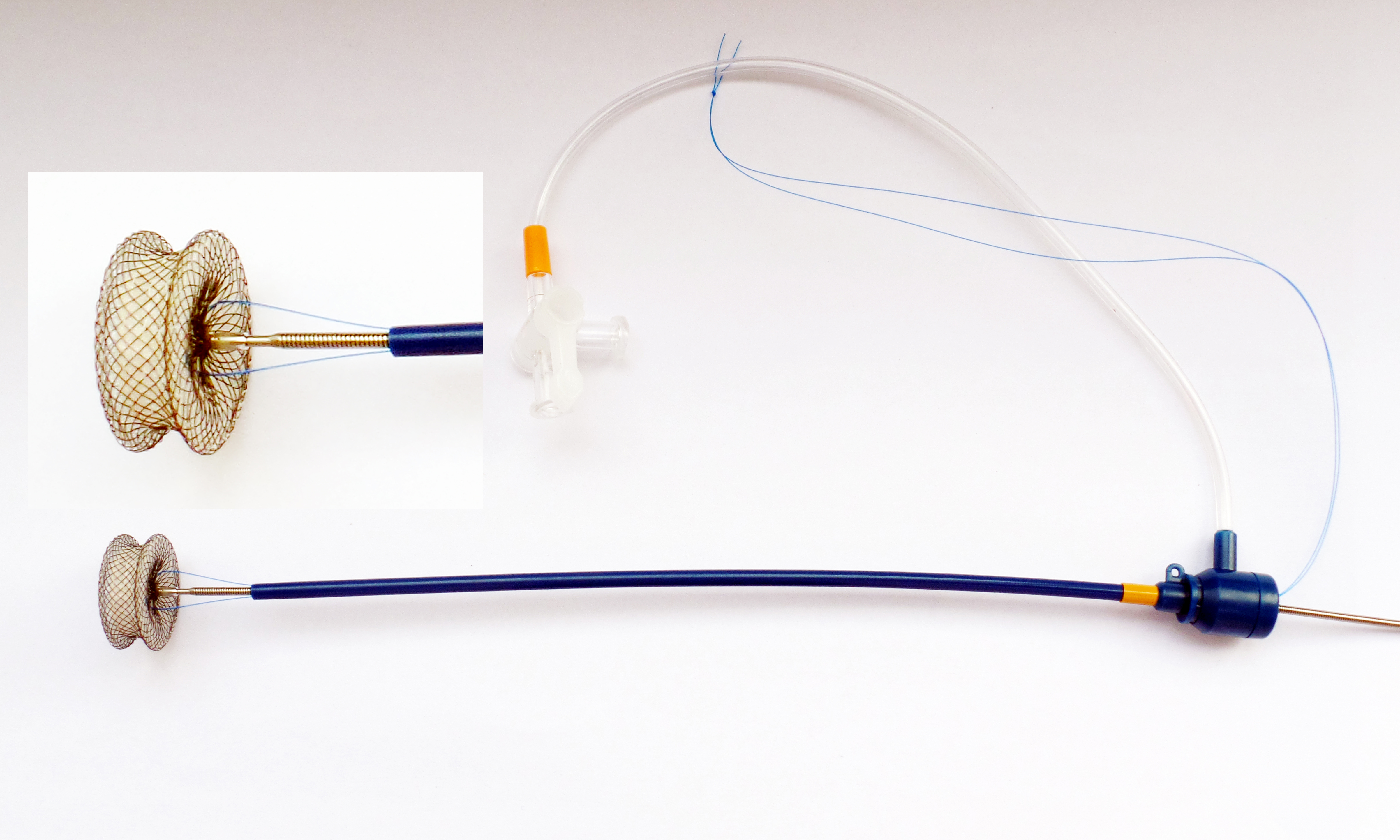

Supplement: Supplementary Figure 1 — The device can be seen attached to the delivery cable and ready to be loaded in the delivery sheath. The polydioxanone stay-in-suture can be seen passed through the center of the device under the screw, then passed back through the sheath. The inset shows the closeup of the device with stay-in-suture. [file Image_1.jpg]
